# The Protein Kinase A Inhibitor KT5720 Prevents Endothelial Dysfunctions Induced by High-Dose Irradiation

**DOI:** 10.3390/ijms25042269

**Published:** 2024-02-14

**Authors:** François-Xavier Boittin, Nathalie Guitard, Maeliss Toth, Diane Riccobono, Hélène Théry, Régis Bobe

**Affiliations:** 1 Unité de Radiobiologie, Département Effets Biologiques des Rayonnements, IRBA—Institut de Recherche Biomédicale des Armées, Place du Général Valérie André, 91223 Brétigny-sur-Orge, France; 2 Université Paris-Saclay, INSERM, Laboratory of Signalling and Cardiovascular Pathophysiology U1180, 91400 Orsay, France; 3 Université Paris-Saclay, INSERM, Hémostase Inflammation Thrombose HITh U1176, 94276 Le Kremlin-Bicêtre, France; regis.bobe@inserm.fr

**Keywords:** endothelial cells, irradiation, apoptosis, ICAM-1, VE-Cadherin, actin cytoskeleton, PKA, KT5720

## Abstract

High-dose irradiation can trigger numerous endothelial dysfunctions, including apoptosis, the overexpression of adhesion molecules, and alteration of adherens junctions. Altogether, these endothelial dysfunctions contribute to the development of tissue inflammation and organ damage. The development of endothelial dysfunctions may depend on protein phosphorylation by various protein kinases, but the possible role of protein kinase A (PKA) has not been investigated so far, and efficient compounds able to protect the endothelium from irradiation effects are needed. Here we report the beneficial effects of the PKA inhibitor KT5720 on a panel of irradiation-induced endothelial dysfunctions in human pulmonary microvascular endothelial cells (HPMECs). High-dose X-irradiation (15 Gy) triggered the late apoptosis of HPMECs independent of the ceramide/P38 MAP kinase pathway or p53. In contrast, the treatment of HPMECs with KT5720 completely prevented irradiation-induced apoptosis, whether applied before or after cell irradiation. Immunostainings of irradiated monolayers revealed that KT5720 treatment preserved the overall integrity of endothelial monolayers and adherens junctions linking endothelial cells. Real-time impedance measurements performed in HPMEC monolayers confirmed the overall protective role of KT5720 against irradiation. Treatment with KT5720 before or after irradiation also reduced irradiation-induced ICAM-1 overexpression. Finally, the possible role for PKA in the development of endothelial dysfunctions is discussed, but the potency of KT5720 to inhibit the development of a panel of irradiation-induced endothelial dysfunctions, whether applied before or after irradiation, suggests that this compound could be of great interest for both the prevention and treatment of vascular damages in the event of exposure to a high dose of radiation.

## 1. Introduction

In addition to hematopoietic syndrome, high-dose irradiation can cause tissue and organ damage, which is partly linked to the deleterious effect of irradiation on the endothelium of blood vessels. Indeed, irradiation can trigger endothelial dysfunctions, leading to impaired tissue/organ perfusion, exacerbated inflammation, and tissue oedema [1,2,3,4]. The mechanisms involved in irradiation-induced tissue inflammation and oedema include the overexpression of adhesion molecules, endothelial cell apoptosis, and the alteration of inter-endothelial cell junctions [1,3,4,5,6,7,8,9]. These processes collectively promote the diapedesis of white blood cells in tissues, leading to the release of pro-inflammatory cytokines and oedema development [1,3,4,5,6,7,8,9].

Numerous pathways involving various protein kinases have been shown to contribute to the development of endothelial dysfunctions, such as apoptosis, the overexpression of adhesion molecules, or the alteration of inter-endothelial junctions in the event of irradiation. Understanding these pathways is crucial to developing more effective therapies to combat endothelial dysfunction.

Irradiation can induce DNA damage in the nucleus, a pathway that can trigger the phosphorylation of p53 by DNA damage kinase, which can lead to the apoptosis of endothelial cells by cytochrome c released from mitochondria (intrinsic pathway) or by the activation of death receptors (extrinsic pathway) [3,4,10,11,12]. However, irradiation can also trigger apoptosis in endothelial cells via another pathway independent of the nucleus and DNA damage, as ceramide generated by the hydrolysis of membrane sphingomyelin by extracellular sphingomyelinases can induce rapid apoptosis via the activation of MAP kinases [4,13,14,15,16].

Irradiation enhances the expression of adhesion molecules, such as ICAM-1, VCAM-1, E-selectin and P-selectin, which facilitates the adhesion of white blood cells to the endothelium and their migration into tissues [1,5,17,18,19]. Pathways involved in the overexpression of adhesion molecules by endothelial cells following irradiation involve the activation of NF-kB and the phosphorylation/activation of protein kinases, such as PKC or JNK in human umbilical vein endothelial cells [18,20,21,22].

At the same time, irradiation can alter the cytoskeleton and cause destabilization and disruption of inter-endothelial adherens junctions made of VE-Cadherin, leading to higher permeability of the endothelial barrier [7,8,23,24,25,26,27]. This process has been linked to MAP kinases and the NF-kB inflammatory pathway in human umbilical vein endothelial cells [8].

Therefore, several protein kinases have been implicated in the development of numerous endothelial dysfunctions post-irradiation in endothelial cell models, suggesting that their inhibition may represent an efficient way to alleviate irradiation-induced endothelial damages. However, the possible role of protein kinase A (PKA) has not been investigated so far. Moreover, suitable pharmacological compounds, capable of preventing the development of a panel of endothelial dysfunctions induced by irradiation, are still missing. Here, we provide evidence that KT5720, a high-affinity ATP-competitive inhibitor of PKA (Ki = 60 nM), effectively prevents or treats a panel of irradiation-induced endothelial dysfunctions in human pulmonary microvascular endothelial cells [28]. Overall, our results suggest that this compound has the potential to reduce both irradiation-induced vascular damage and associated organ injury.

## 2. Results

### 2.1. Effects of Sphingosine-1-P, p38 MAP Kinase, or p53 Inhibitors on Irradiation-Induced Apoptosis of HPMECs

**Figure 1:**
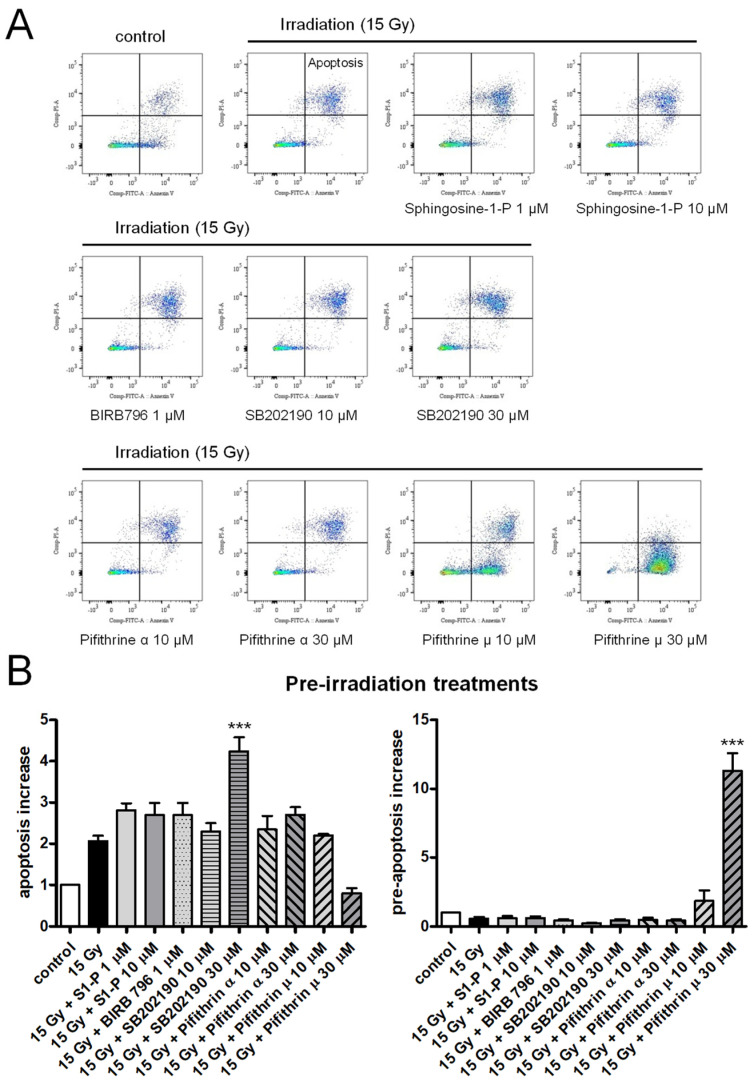
Effects of sphingosin-1-Phosphate and p38 MAP kinase or p53 inhibitors on irradiation (15 Gy)-induced apoptosis in HPMECs. (**A**) Flow cytometry analysis of HPMECs apoptosis in control, after high-dose irradiation (15 Gy), and in irradiated HPMECs pre-treated (1 h before irradiation) with sphingosine-1-Phosphate (1 and 10 µM), BIRB796 (1 µM), SB202190 (10 and 30 µM), or Pifithrin α and µ (10 and 30 µM). Apoptosis measurements were performed 72 h after irradiation. Quadrants drawn on pictures allow us to measure the proportion of apoptotic and pre-apoptotic cells among HPMECs populations (Annexin V+/PI+ and Annexin V+/PI− cells, respectively). The color scale of graphs (blue, green and red) is linked to the number of events (cells) recorded (blue: isolated events, green and red: superimposed events). (**B**) Average increases in the proportion of apoptotic cells after high-dose irradiation (15 Gy), and in irradiated HPMECs pre-treated with sphingosine-1-Phosphate (S1-P, 1 and 10 µM), BIRB796 (1 µM), SB202190 (10 and 30 µM), or Pifithrin α and µ (10 and 30 µM). Data on graphs represent the average of 3 experiments (*** *p* < 0.001).

Apoptosis was assessed in HPMECs 72 h after irradiation, using an FITC Annexin V/Propidium Iodide apoptosis detection kit. As evidenced by the significant increased fraction of apoptotic cells, X-irradiation at a high dose (15 Gy) induced the late apoptosis of HPMECs, as previously reported (Figure 1) [29]. In endothelial cells, apoptotic pathways activated by irradiation may involve ceramide synthesis through membrane sphingomyelinase activation, p38MAP kinase, or p53 activation, which results in caspase activation and cell death [4,12,13,14,15,16].

To investigate which apoptotic pathway may be involved in HPMECs, the effects of sphingosine-1-P (an antagonist of the ceramide pathway) as well as p38 MAP kinases and p53 inhibitors pre-treatment have been tested on the irradiation-induced apoptosis of HPMECs [13,30,31,32,33,34]. As shown in Figure 1, even a high concentration of sphingosine-1-P (10 µM) did not significantly reduce irradiation-induced apoptosis in HPMECs [13]. The p38MAP kinase inhibitors SB202190 and BIRB796 also failed to inhibit irradiation-induced apoptosis in HPMECs (Figure 1) [33,34]. Finally, the p53 inhibitors Pifithrin α and µ, that block p53-dependent transcriptional activity and p53 binding to mitochondria, respectively, did not protect HPMECs from irradiation-induced apoptosis [30,31,32]. The p53 inhibitor Pifithrin µ appeared to worsen irradiation-induced pre-apoptosis as a clear increase in the fraction of Annexin V+ cells was observed (Figure 1).

### 2.2. Both Pre- and Post-Irradiation Treatments with KT5720 Prevent Irradiation-Induced Apoptosis of HPMECs

**Figure 2:**
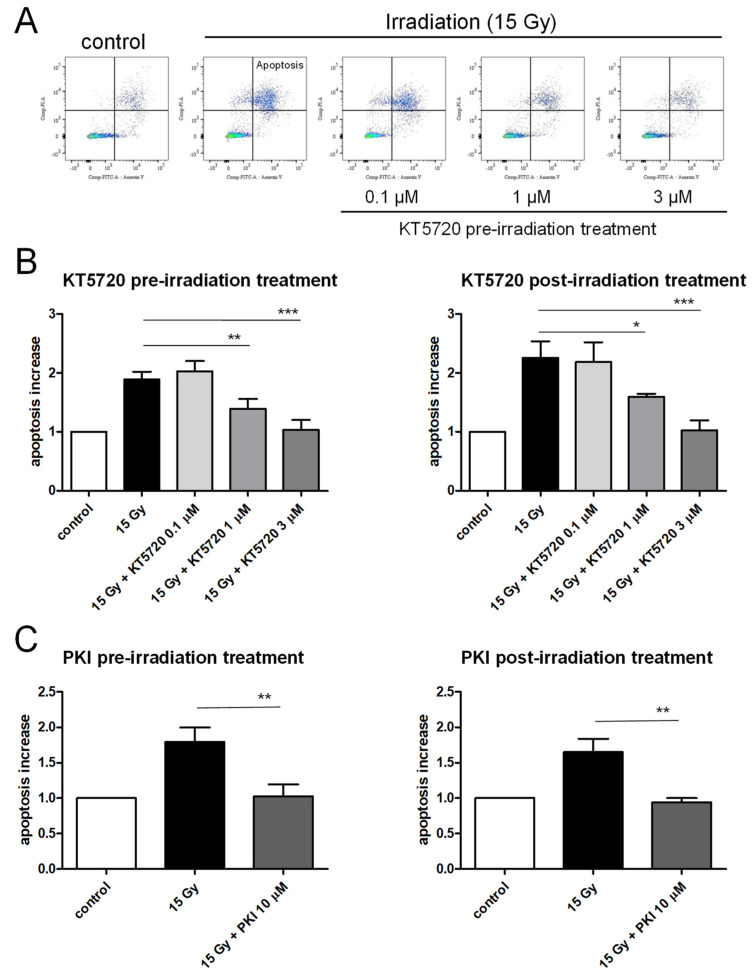
Effects of pre- and post-irradiation treatments with KT5720 or PKI 14–22 amide (PKI) on irradiation (15 Gy)-induced apoptosis in HPMECs. (**A**) Flow cytometry analysis of HPMECs apoptosis in control, after high-dose irradiation (15 Gy), and in irradiated HPMECs pre-treated with KT5720 (0.1, 1, and 3 µM). Apoptosis measurements were performed 72 h after irradiation. Quadrants drawn on pictures allow us to measure the proportion of apoptotic cells (Annexin V+/PI+) among HPMECs populations. The color scale of graphs (blue, green and red) is linked to the number of events (cells) recorded (blue: isolated events, green and red: superimposed events). (**B**) Average increases in the proportion of apoptotic cells after high-dose irradiation (15 Gy) and in irradiated HPMECs treated with KT5720 before (1 h) or after (1 h) irradiation. Data on graphs represent, respectively, the average of 5 and 4 experiments for pre- and post-irradiation treatments with KT5720 (* *p* < 0.05, ** *p* < 0.01, *** *p* < 0.001). (**C**) Average increases in the proportion of apoptotic cells after high-dose irradiation (15 Gy) and in irradiated HPMECs treated with PKI before (1 h) or after (1 h) irradiation. Data on graphs represent, respectively, the average of 4 and 3 experiments for pre- and post-irradiation treatments with PKI (** *p* < 0.01).

In contrast to the lack of effect of Sphingosine-1-P, p38 MAP kinases, or p53 blockers, the pre-treatment of HPMECs with the protein kinase A inhibitor KT5720 was effective at blocking irradiation-induced apoptosis (Figure 2). Notably, protection was achieved even at a low concentration of 1 µM, with 3 µM concentration providing even stronger protection (as shown in Figure 2A,B) [35]. The protective effect of KT5720 against irradiation-induced apoptosis was dose-dependent, but most importantly, this compound was efficient to blunt irradiation-induced apoptosis even when applied after exposure (Figure 2B). In fact, the efficiency of KT5720 to counteract irradiation-induced apoptosis remained the same regardless of whether it was applied before or after radiation exposure. Similar results were observed using another PKA inhibitor (PKI 14–22 amide (PKI)) [36]. Indeed, pre- or post-irradiation treatments of HPMECs with PKI 10 µM completely abolished irradiation-induced apoptosis (Figure 2C).

### 2.3. KT5720 Post-Irradiation Treatment Prevents Irradiation-Induced Alteration of Endothelial Monolayers

**Figure 3:**
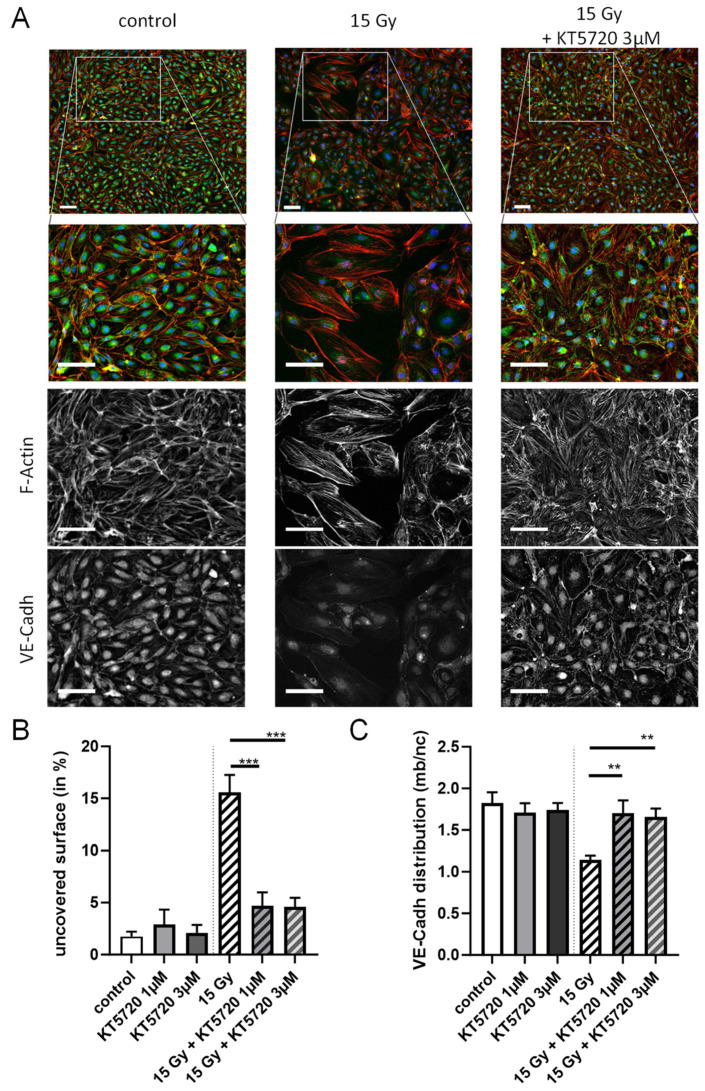
Effects of KT5720 on irradiation-induced alteration of the actin cytoskeleton and VE-Cadherin adherens junctions of confluent endothelial monolayers. (**A**) Merged images represent staining of F-actin (red) and VE-Cadherin (VE-Cadh, green) in HPMECs monolayers in control (left column) and 72 h after irradiation at high dose (15 Gy) with or without post-irradiation treatment with KT5720 (right and middle columns, respectively). Cell nucleus were stained with DAPI (blue) while yellow color in merged images indicates colocalization of F-actin (red) and VE-Cadherin (green). In each column, a merged wide field image is represented on top, while a magnification of an area drawn on the image is represented below. Single staining corresponding to the selected area is also represented in gray levels under the merged image. Images are representative of 3 experiments. Scale bar: 100 µM. (**B**) The bar graph represents the percentage of surface not covered by cells in endothelial monolayers (uncovered surface) in control (with or without KT5720 treatment (1 and 3 µM)) and in irradiated endothelial monolayers treated or not with KT5720 after irradiation. (**C**) The bar graph represents averaged ratio of VE-Cadherin staining fluorescence intensities between the plasma membrane and the nucleus area (VE-Cadherin distribution) in control (with or without KT5720 treatment (1 and 3 µM)) and in irradiated endothelial monolayers treated or not with KT5720 after irradiation. Data on graphs represent the average of 3 experiments (** *p* < 0.01, *** *p* < 0.001).

To examine the long-term impacts of high-dose irradiation (15 Gy) on the actin cytoskeleton and adherens junctions of confluent endothelial monolayers, HPMECs were stained with both Phalloidin and an anti-VE-Cadherin antibody 72 h after irradiation (Figure 3). While the non-irradiated endothelial monolayer (control) appears rather homogeneous and continuous with rare intercellular spaces, high-dose-irradiated endothelial monolayers exhibited extensive areas of missing cells (Figure 3A). As a consequence, the percentage of surface not covered by cells (uncovered surface) was significantly increased in irradiated endothelial monolayers compared to the control (Figure 3B). Importantly, the post-irradiation treatment of endothelial monolayers with KT5720 (1 and 3 µM) significantly reduced the uncovered surface to values similar to the non-irradiated control. This suggests that the compound offers protection against the detrimental effects of irradiation on the arrangement of endothelial monolayers. At the cellular level, high-dose irradiation (15 Gy) also caused a rearrangement of the actin cytoskeleton and the adherens junctions made of VE-Cadherin. In control cells, the actin cytoskeleton was localized mostly near the plasma membrane with low cytosolic staining corresponding to actin stress fibers. In contrast, a bright near-plasma membrane actin staining with highly stained cytosolic stress fibers was observed in high-dose-irradiated HPMECs (Figure 3A). An alteration of VE-cadherin staining was also observed in high-dose-irradiated HPMECs. Indeed, while plasma membrane VE-Cadherin staining was bright and continuous in non-irradiated cells, it becomes irregular, weak, or even absent in high-dose-irradiated HPMECs (Figure 3A). In support of these observations, the ratio between plasma membrane and nucleus area VE-Cadherin staining intensities was significantly decreased in irradiated endothelial monolayers, suggesting that the plasma membrane expression of VE-Cadherin is decreased in HPMECs 72 h after irradiation (Figure 3C). In this case, plasma membrane VE-Cadherin may have been degraded and/or displaced from the plasma membrane toward the cytosol/nucleus area.

Post-irradiation treatment with KT5720 (1 and 3 µM) did not prevent the impact of irradiation on the actin cytoskeleton in HPMECs. We observed numerous actin stress fibers in the cytosol of irradiated HPMECs treated with KT5720 (Figure 3A). However, the brightness and regularity of VE-Cadherin staining on the plasma membrane of irradiated HPMECs were preserved when cells were treated with KT5720 compared to cells that were solely irradiated (Figure 3A). Plasma membrane VE-Cadherin staining of irradiated endothelial cells treated with KT5720 appeared similar to the staining of non-irradiated cells. In accordance with these observations, the ratio between plasma membrane and nucleus area VE-Cadherin staining intensity of KT5720-treated irradiated endothelial monolayers was not significantly different from non-irradiated cells (Figure 3C). Overall, these results indicate that a decrease in VE-Cadherin plasma membrane expression after irradiation is prevented by KT5720 treatment. Therefore, this compound has the potential to protect VE-Cadherin-composed adherens junctions or facilitate their reconstitution following irradiation.

### 2.4. Effects of KT5720 Treatment and Irradiation on Impedance of HPMEC Monolayers

**Figure 4:**
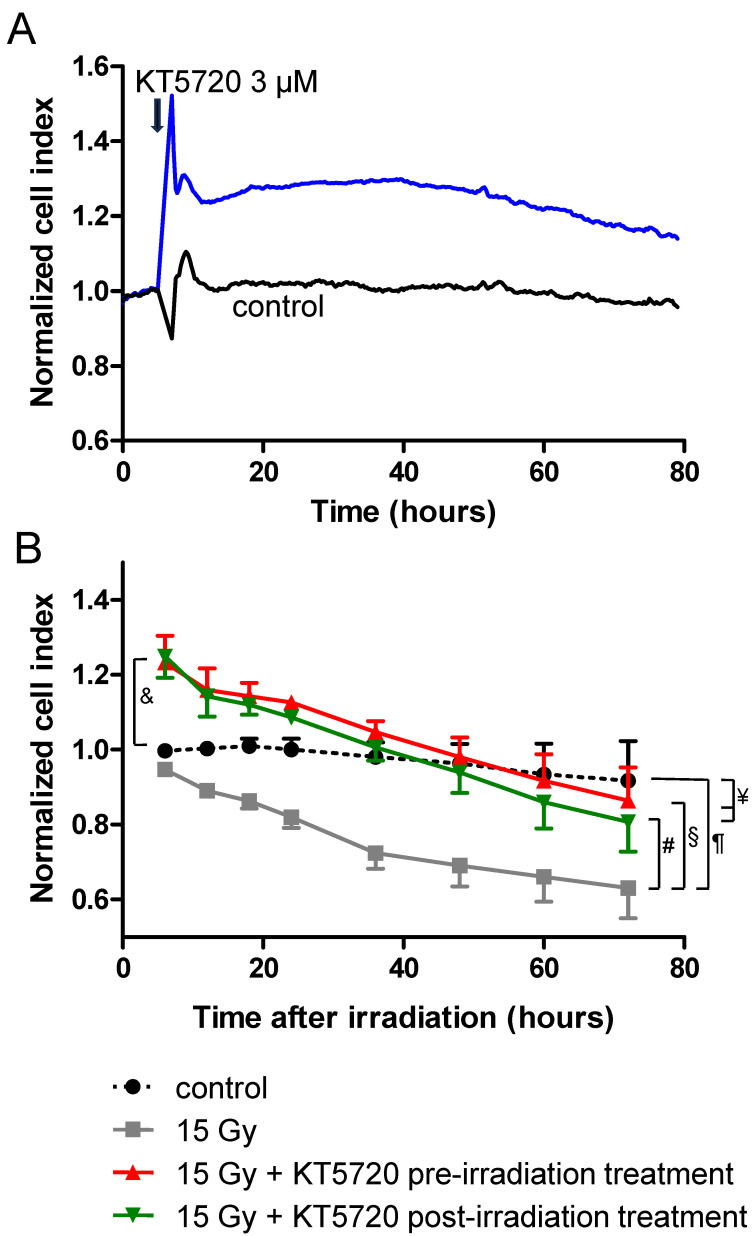
Effect of KT5720, irradiation, and both on cell index of confluent HPMEC monolayers measured using a xCELLigence cell analyzer. (**A**) Representative recordings of normalized cell index in HPMEC monolayers treated or not with KT5720 (3 µM). The arrow on the graph indicates the time of KT5720 addition. Data are representative of 3 experiments. (**B**) Averaged normalized cell index values from 6 to 72 h after irradiation in irradiated HPMECs monolayers treated or not with KT5720 before or after irradiation. Average normalized cell index values of corresponding non-irradiated HPMEC monolayers (control) are represented on the same graph. Data on graph represent averaged values from 3 experiments. # and §: Averages values for 15 Gy + KT5720 pre- and post-irradiation treatment are significantly higher than 15 Gy average values from 6 to 72 h after irradiation (from *p* < 0.05 to *p* < 0.001 depending on time after irradiation); ¶: Average values for 15 Gy are significantly lower than control from 36 to 72 h after irradiation (from *p* < 0.05 to *p* < 0.01 depending on time after irradiation); ¥: Averages values for 15 Gy + KT5720 pre- and post-irradiation treatment are not significantly different from control values from 12 to 72 h after irradiation. &: Averages values for 15 Gy + KT5720 pre- and post-irradiation treatment are significantly higher than control values 6 h after irradiation.

Impedance of confluent HPMEC monolayers was evaluated by measuring the cell index values of monolayers using a xCELLigence cell analyzer. As illustrated in Figure 4A, the treatment of HPMECs with KT5720 (3 µM) induced a rapid and sustained increase in impedance for at least 72 h. In confluent monolayers, such rapid increases in impedance are likely due to the strengthening of inter-endothelial junctions and/or changes in cell morphology [37], rather than cell proliferation. In contrast, when compared to non-irradiated cells, the high-dose (15 Gy) irradiation of HPMEC monolayers triggered a slow decrease in impedance from ~6 h after irradiation (Figure 4B). This slow and regular decrease in impedance induced by irradiation may result from the apoptosis of a fraction of endothelial cells, but also from the alteration of inter-endothelial junctions causing gaps within the monolayer (Figure 2 and Figure 3). For irradiated HPMEC monolayers treated with KT5720 (before or after irradiation), the average impedance values measured between 6 and 72 h after irradiation were significantly enhanced compared to monolayers that were solely irradiated. Just after irradiation (6 h), the average impedance values of irradiated HPMECs monolayers treated with KT5720 were also significantly higher than non-irradiated monolayers. These observations may be explained by the sustained increase in impedance caused by KT5720 alone, and by a protective effect against irradiation-induced endothelial damages.

### 2.5. Both Pre- and Post-Irradiation Treatments with KT5720 Reduce Irradiation-Induced ICAM-1 Overexpression in HPMECs

**Figure 5:**
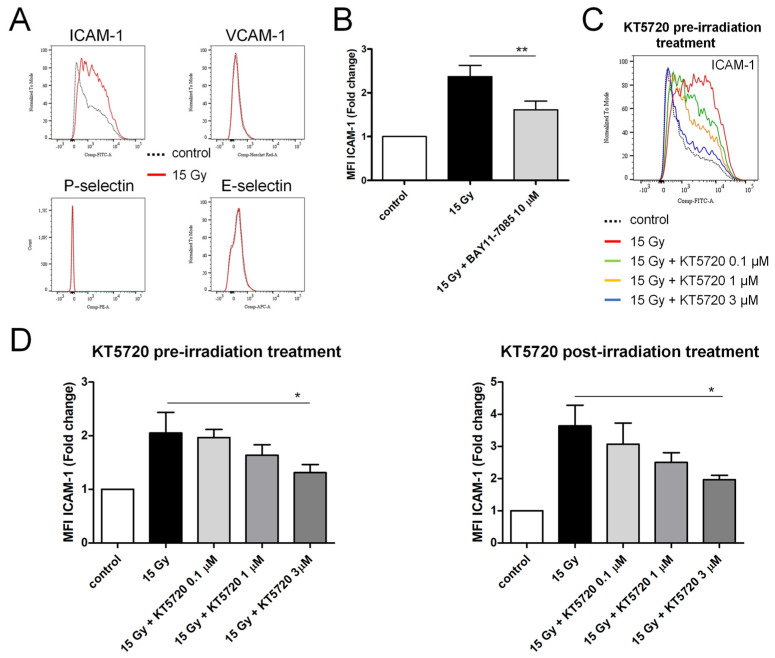
Effect of pre- and post-irradiation treatments with KT5720 on irradiation (15 Gy)-induced ICAM-1 overexpression in HPMECs. Membrane expression of 4 adhesion molecules (ICAM-1, E- and P-Selectin, VCAM-1) was measured using flow cytometry 24 h after 15 Gy-irradiation. (**A**) Merged histograms of ICAM-1, E- and P-Selectin, and VCAM-1 staining in control and 15 Gy-irradiated HPMECs. (**B**) Average normalized MFI values obtained for ICAM-1 staining in irradiated HPMECs treated or not with BAY11-7085 (10 µM). Data on graph represent the average of 5 experiments. (**C**) Merged histograms of ICAM-1 staining in control and 15 Gy-irradiated HPMECs treated or not with KT5720 (0.1, 1, and 3 µM) 1 h before irradiation. (**D**) Average normalized MFI values obtained for ICAM-1 staining in irradiated HPMECs treated or not with KT5720 1 h before (left: pre-irradiation treatment) or after (right: post-irradiation treatment) irradiation. Data on graphs represent, respectively, the average of 4 and 3 experiments for pre- and post-irradiation treatments with KT5720 (* *p* < 0.05, ** *p* < 0.01).

Confluent HPMECs were irradiated at a high dose (15 Gy). After 24 h, cells were detached, fixed, and the membrane expression levels of adhesion molecules (ICAM-1, VCAM-1, E-selectin, P-selectin) were measured using flow cytometry. The membrane expression level of ICAM-1 increased 24 h after irradiation, while it remained unchanged for the other adhesion molecules (Figure 5A,B). The preincubation of HPMECs with BAY11-7085, an inhibitor of NF-κB, reduced the overexpression of ICAM-1 induced by irradiation, suggesting the involvement of this transcription factor in the effect of irradiation (Figure 5B) [38]. Furthermore, the overexpression of ICAM-1 induced by irradiation was dose-dependently reduced by treatment with KT5720 before and after irradiation (Figure 5C,D), yet the impact of KT5720 was only significant for a concentration of 3 µM.

### 2.6. Measurement of PKA Activity in Irradiated HPMECs

**Figure 6:**
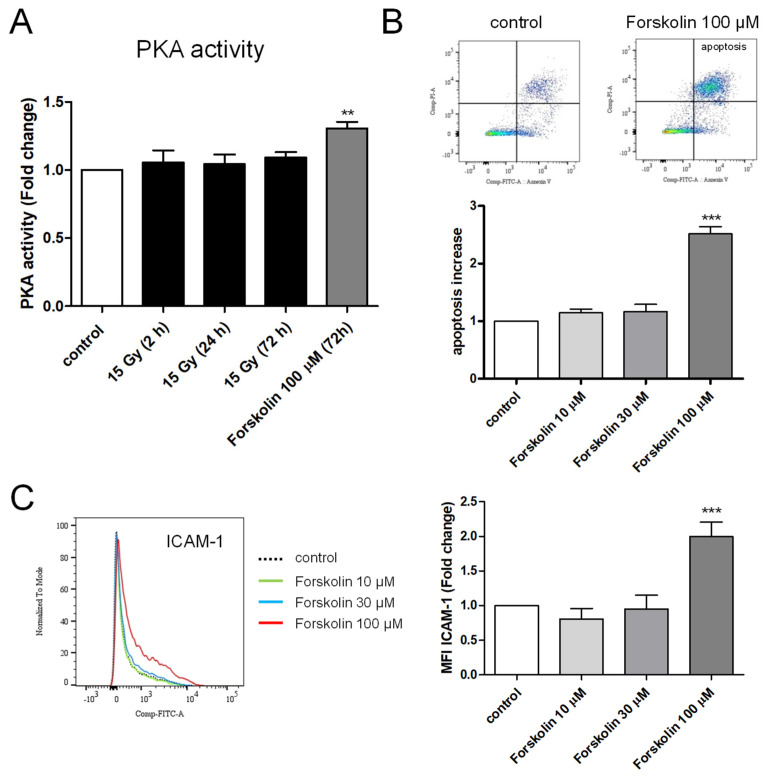
PKA activity and effect of Forskolin on apoptosis and ICAM-1 membrane expression in HPMECs. (**A**) PKA activity measured in control and irradiated HPMECs (2, 24, and 72 h after irradiation). Data represent the average of 3 experiments. The effect of Forskolin (100 µM, 72 h) on PKA activity is represented on the same graph. (**B**) Top: Effect of Forskolin (100 µM) on apoptosis of HPMECs 24 h after its addition. The color scale of graphs (blue, green and red) is linked to the number of events (cells) recorded (blue: isolated events, green and red: superimposed events). Below: Average increases in the proportion of apoptotic cells after Forskolin (10, 30, or 100 µM) treatment of HPMECs. Data on graph represent the average of 3 experiments (*** *p* < 0.001). (**C**) Left: Merged histograms of ICAM-1 staining in control and in HPMECs treated with Forskolin (10, 30, or 100 µM) for 24 h. Right: Average normalized MFI values obtained for ICAM-1 staining in Forskolin-treated HPMECs. Data on graphs represent the average of 4 experiments (** *p* < 0.01, *** *p* < 0.001).

PKA activity was measured in whole-cell extracts from control and high-dose-irradiated HPMECs 2, 24, and 72 h after irradiation. As shown in Figure 6A, the PKA activity of HPMECs was not significantly modified by high-dose irradiation whatever the time after irradiation. In contrast, PKA activity was significantly increased by Forskolin, a cell-permeant activator of adenylate cyclase, leading to an increased cAMP level and the activation of PKA [39].

### 2.7. Effects of Forskolin on Apoptosis and ICAM-1 Membrane Expression in HPMECs

To further address the role for PKA, we analyzed the effects of Forskolin treatment in HPMECs. As shown in Figure 6B, the treatment of HPMECs with Forskolin (100 µM) induced significant apoptosis in confluent HPMECs, as evidenced by the significant increase in the fraction of Annexin V+/PI+ cells.

Additionally, Forskolin (100 µM) treatment induced membrane ICAM-1 overexpression, without a significant effect on the expression of the other adhesion molecules investigated (Figure 6C).

## 3. Discussion

In order to find possible countermeasures targeting the endothelium and preventing irradiation-induced organ damages, we examined here the effect of the protein kinase A inhibitor KT5720 on a panel of irradiation-induced endothelial dysfunctions in human pulmonary microvascular endothelial cells. We first show that high-dose irradiation (15 Gy) induced late apoptosis in confluent HPMECs. Such irradiation-induced late death has already been described in proliferating human microvascular endothelial cells and was attributed to DNA-damage-induced mitotic death, while early apoptosis was shown to involve the ceramide pathway and P38 MAP kinase [13,14,15]. In agreement with these observations, irradiation-induced late apoptosis was not affected by high concentrations of sphingosin-1-Phosphate (an antagonist of the ceramide apoptosis pathway) or by p38 MAP kinase inhibitors, suggesting that these pathways were not involved [13,14,15,40]. The irradiation-induced late apoptosis of HPMECs was also not linked to p53 activation, as potent blockers of the p53 pathway (Pifithrin α and µ) remained inefficient. In endothelial cells, the role of p53 in radiation-induced apoptosis is in fact rather controversial as in vivo studies have demonstrated that p53 protects endothelial cells from irradiation, while other studies have shown that the p53 pathway is involved in irradiation-induced apoptosis [10,11]. Altogether, our results indicate that late irradiation-induced apoptosis in confluent HPMECs does not involve ceramide-, p38 MAP kinase-, nor p53-dependent pathways.

In contrast, both pre- and post-irradiation treatments with the protein kinase A inhibitor KT5720 potently prevented the apoptosis of HPMECs in a dose-dependent manner. Such an anti-apoptotic effect of KT5720 has been reported in experimental necrotizing enterocolitis, where it reduces the bacteria-induced apoptosis of intestinal epithelial cells [35]. Interestingly, the ability of KT5720 to protect HPMECs from irradiation-induced apoptosis was the same whether applied before or after irradiation. Due to its anti-apoptotic effect, KT5720 significantly protected endothelial monolayers from obvious deleterious irradiation effects, since areas missing cells were significantly reduced in endothelial monolayers that had been treated after irradiation.

In HPMEC monolayers, irradiation at a high dose (15 Gy) also induced the formation of numerous actin stress fibers and altered the adherens junctions, as attested by the modification of VE-Cadherin distribution observed 72 h after irradiation. In irradiated HPMEC monolayers treated with KT5720, VE-Cadherin distribution was not significantly different from non-irradiated cells, suggesting that KT5720 protects adherens junctions made of VE-Cadherin from irradiation. Another possible explanation may be that this compound stimulates VE-Cadherin expression and localization at the plasma membrane after irradiation.

Surprisingly, KT5720 treatment rapidly increases the impedance of HPMEC monolayers, suggesting that KT5720 may either strengthen endothelial junctions or alter cell morphology [37]. We did not notice an obvious change in cell morphology in KT5720-treated cells, but as phosphorylation by PKA can activate Phosphodiesterase 4 (PDE4) in endothelial and smooth muscle cells, the effect of the PKA inhibitor KT5720 on monolayer impedance could be linked to PDE4 inhibition and a subsequent rise in cAMP concentration, which is indeed known to reinforce endothelial junctions and increase the transendothelial electrical resistance of endothelial monolayers [41,42,43,44].

The decrease in impedance induced by high-dose irradiation may be explained by the loss of cells linked to apoptosis, and/or by the weakening of adherens junctions between endothelial cells, as suggested by the significant change in the distribution of VE-cadherin observed. The decrease in impedance induced by irradiation was significantly reduced by treatment with KT5720, whatever the time elapsed after irradiation. This effect of KT5720 may be explained by its protective action against apoptosis-related loss of monolayer cells but also by its protecting role on endothelial adherens junctions.

The beneficial effects of KT5720 also include the reduction in the irradiation-induced NF-kB-dependent membrane overexpression of ICAM-1. As observed for apoptosis, both pre- and post-irradiation treatments of HPMECs with KT5720 reduced the irradiation-induced overexpression of ICAM-1.

Altogether, these results indicate that the protein kinase A inhibitor KT5720 can protect HPMECs against a range of irradiation-induced endothelial dysfunctions, suggesting a possible role for PKA in the onset of irradiation-induced effects. This was further supported by the fact that another PKA inhibitor, PKI 14–22 amide, mimics the protective effect against irradiation-induced apoptosis. Nonetheless, PKA activity in whole-cell extracts from HPMECs did not change 2, 24, and 72 h after irradiation, suggesting that a global increase in PKA activity is unlikely to be a trigger for the development of irradiation-induced endothelial dysfunctions. However, one cannot exclude that transient PKA activation occurs at a different time (for example between 2 h and 24 h), and also that the PKA colorimetric assay used with whole-cell extracts may not be sensitive enough to measure localized changes in PKA activity [45]. The fact that forskolin (an activator of adenylate cyclase that increases cytosolic cAMP and PKA activity) can mimic some effects of irradiation on apoptosis or ICAM-1 overexpression suggests that phosphorylation by PKA may occur in the pathways triggered by irradiation, leading to endothelial dysfunctions [39]. However, some effects of KT5720 reported in this study may also be explained by an inhibition of other protein kinases, as this compound is not a strictly specific inhibitor of PKA [46,47]. To obtain the answer to this question, further investigation is required.

Overall, our results indicate that KT5720 can prevent the development of a range of endothelial dysfunctions induced by high-dose irradiation in HPMECs. Importantly, the efficacy of KT5720 was the same whether applied 1 h before or 1 h after irradiation, suggesting that this compound may be of interest for the prevention and/or the emergency treatment of irradiation-induced vascular and organ damages.

## 4. Materials and Methods

### 4.1. Materials

KT5720 (PKA inhibitor), PKI 14–22 amide, p53 inhibitors (Pifithrin α and µ), p38 MAP kinases inhibitors (BIRB796 and SB202190), Sphingosine-1-P, Forskolin, and the NF-κB inhibitor BAY11-7085 were all purchased from Tocris (Bristol, UK). For immunostainings, Phalloidin 547 (FP-AZ0330) was from FluoProbes, Interchim, (Montluçon, France), while the FITC-conjugated mouse anti-VE-Cadherin (CD144) antibody (clone 55-7H1, cat no. 580411) was from BD Pharmingen (Franklin Lakes, NJ, USA). The mounting medium (DAPI Fluoromount-G^TM^ (cat no. 0100-20)) was purchased from Southern Biotech (Birmingham, AL, USA). For flow cytometry analysis, all antibodies (BB515 mouse anti-human CD54 (ICAM-1, cat no. 564685), BUV737 mouse anti-human CD106 (VCAM-1, cat no. 565418), PE mouse anti-human CD62P (P-Selectin, cat no. 555524), APC mouse anti-human CD62E (E-Selectin, cat no. 551144)), and the FITC Annexin V/Propidium Iodide apoptosis detection kit were obtained from BD Biosciences (Franklin Lakes, NJ, USA).

### 4.2. Culture of Microvascular Endothelial Cells

Human pulmonary microvascular endothelial cells (HPMECs) were purchased from Promocell (Heidelberg, Germany) and cultured either in T25 flasks, 6-well plates, 8-chamber Lab-Tek, or E-plates L16 (Acea Biosciences, San Diego, CA, USA). To improve cell adherence, T25 flasks, 6-well plates, or 8-chamber Lab-Tek slides were coated with attachment factor (Gibco, Waltham, MA, USA). HPMECs were cultured using endothelial cell growth medium MV2 supplemented with endothelial cell growth medium supplement MV2 (Promocell) and Gentamicin/amphotericin (Gibco). All experiments were performed once the confluence of endothelial cells was reached. HPMECs from passage 3 to 7 were used in all experiments.

### 4.3. Irradiation and Treatments

Confluent HPMECs cultured either in 6-well plates, 8-chamber Lab-Tek slides, or E-plates L16 (Acea Biosciences) were irradiated using a SARRP X-irradiator (Xstrahl, Walsall, UK) at a dose of 15 Gy, with a dose-rate value of 0.68 Gy/min. To ensure that the irradiation dose was accurate, dosimetry was performed as previously described [29,48].

In all experiments, the protein kinase A inhibitor KT5720 was applied either 1 h before irradiation (pre-irradiation treatment) or 1 h after irradiation (post-irradiation treatment). In the experiments from Figure 1, HPMECs were pre-treated with the pharmacological compounds 1 h before being irradiated.

### 4.4. Immunostainings

For immunostainings, HPMECs were cultured in 8-chamber Lab-Tek slides. After being washed with DPBS, HPMECs were fixed with DPBS containing 2% paraformaldehyde for 15 min and then permeabilized with Triton X-100 (0.2%) for 3 min at room temperature. In order to avoid the non-specific binding of antibodies, HPMECs were incubated with DPBS containing 5% of bovine serum albumin for 1 h at room temperature. HPMECs were then stained in DPBS containing 5% of bovine serum albumin, an FITC-conjugated mouse anti-CD144 (VE-Cadherin) antibody (diluted 1:5), and Phalloidin 547 (diluted 1:250) for 45 min at room temperature. Stained HPMECs were extensively washed with DPBS before mounting the coverslips on glass slides using DAPI Fluoromount-G^TM^. Fluorescence images were acquired using a MICA Microhub microscope (Leica, Wetzlar, Germany) and LAS-X V2021, 2.0 software. The percentage of surface not covered by cells in endothelial monolayers (uncovered surface) was measured in 6 to 12 images from each experiment using Image J 1.53c software. In order to evaluate VE-Cadherin localization (membrane or nucleus area), the ratio of maximal fluorescence intensities at the plasma membrane over mean of fluorescence intensities in the nucleus area was calculated from the fluorescence profiles of VE-Cadherin-FITC staining in endothelial cells (generated by Image J software). Measurements were performed on 15–25 fluorescence profiles from 3 to 5 images in each condition.

### 4.5. Apoptosis Measurements

The apoptosis of HPMECs cultured in 6-well plates was evaluated using an FITC Annexin V/Propidium Iodide apoptosis detection kit (BD Biosciences). Using detach kit 125 (Promocell), HPMECs were detached and centrifugated for 5 min. Cells were washed twice with cold PBS according to the manufacturer’s instructions. HPMECs were then resuspended in 100 μL binding buffer and stained for 15 min with 5 μL FITC-Annexin V and 5 μL Propidium Iodide (PI) at room temperature. After the further addition of binding buffer, the apoptosis of HPMECs was measured rapidly using a Becton Dickinson LSR II cytometer. Analysis was performed using FlowJo V10 software, which allows for the proportions of pre-apoptotic cells (PI−, FITC Annexin V+), apoptotic cells (PI+, FITC Annexin V+), necrotic cells (PI+, FITC Annexin V−), and normal cells (PI−, FITC Annexin V−) to be defined.

### 4.6. Measurements of Endothelial Cell Surface Expression of Adhesion Molecules

Flow cytometry was used to evaluate the membrane expression of adhesion molecules (ICAM-1, VCAM-1, P-selectin, and E-selectin) in HPMECs following irradiation and/or pharmacological treatments. HPMECs cultured in 6-well plates were detached using detach kit 125 (Promocell), centrifugated, and then fixed in PBS containing 1% paraformaldehyde for 30 min. After extensive washing in PBS, HPMECs were stained for 30 min with a panel of antibodies, including BB515 mouse anti-human CD54 (ICAM-1), BUV737 mouse anti-human CD106 (VCAM-1), PE mouse anti-human CD62P (P-selectin), and APC mouse anti-human CD62E (E-selectin). After being stained, HPMECs were extensively washed with PBS (3×). A Becton Dickinson LSR II flow cytometer was then used to analyze the fluorescence of HPMECs. Fluorescence compensations were set in each experiment using an anti-mouse Ig, κ/Negative control compensation particles set (BD Biosciences). Data were analyzed with the FlowJo V10 software, allowing us to measure the Median Fluorescence Intensity (MFI) for ICAM-1, VCAM-1, P-selectin, and E-selectin. MFI values allowed us to compare the cell surface expression of ICAM-1, P-selectin, E-selectin, and VCAM-1 in control and after cell irradiation/treatment.

### 4.7. Measurements of Impedance in HPMEC Monolayers

The impedance of confluent HPMEC monolayers was continuously monitored using a RTCA xCELLigence cell analyzer (Acea Biosciences Inc.). This analyzer allowed us to record the dimensionless parameter cell index (proportional to impedance), which is influenced by cell adhesion, cell growth, cell morphology, and also by cell junctions. HPMECs were seeded in E-plates L16 (Acea Biosciences) at a density of 5000 cells/well, placed in the RTCA iCELLigence instrument, and then incubated at 37 °C/5% CO_2_ for impedance measurements. After confluence was reached with stable cell index, HPMECs were irradiated at a high dose (15 Gy) and/or treated with the PKA inhibitor KT5720. In each individual experiment, measurements were performed in duplicate. Data are represented as average normalized cell index values before and at different times after cell irradiation.

### 4.8. Measurements of PKA Activity in HPMECs

A PKA colorimetric activity kit (EIAPKA, Thermofisher Scientific, Waltham, MA, USA) was used to measure PKA activity in whole-cell extracts at different times following high-dose irradiation (15 Gy). Samples were prepared in cell lysis buffer according to the manufacturer’s instructions 2, 24, and 72 h after the irradiation of HPMECs. The PKA activity of irradiated HPMECs was reported to the activity measured in non-irradiated cells. The effect of Forskolin on PKA activity was also investigated in the same cells.

### 4.9. Statistical Analysis

In all bar charts, data are expressed as means ± SEM. Statistical analyses were conducted using either *t*-tests or ANOVA tests with GraphPad Prism V10.1.2 (324) software (San Diego, CA, USA). The number of experiments performed together with statistical significance is indicated in the figure legends.

## Data Availability

Data is contained within the article.
